# Orthopantomography Detection of Atheroma Plaques and Its Relationship with Periodontal Disease and Missing Teeth

**DOI:** 10.1155/2024/8873720

**Published:** 2024-03-04

**Authors:** Rodrigo Quevedo García, Sara Arnaiz Díez, Esteban Pérez Pevida, María Lourdes Del Río Solá

**Affiliations:** ^1^Faculty of Dentistry, European University Miguel de Cervantes, Valladolid, Spain; ^2^Burgos University Hospital, Burgos, Spain; ^3^Vascular Surgery Department, Valladolid University Hospital, Valladolid, Spain

## Abstract

**Background:**

The aim of this study is to determine the atheromatous plaques' prevalence in orthopantomography and their relationship with periodontal disease and missing teeth. *Material and Methods*. Orthopantomographs of 1,254 patients over 18 years of age from Clínica Arlanza in Lerma, Burgos, were examined between 2017 and 2021. A Planmeca ProOne® orthopantomograph (68 kV, 7 mA, and 10 sg) was used. Statistical analysis was carried out using SPSS Statistics® version 25. The results of the categorical variables were described as frequencies (%). Contingency tables were made with the qualitative variables, and the chi-square test was applied to study the relationship among them. The measure of statistical power used was the relative risk (RR), which was described with its respective 95% confidence interval (CI). Student's *t*-test was applied to study the relationship between the qualitative variable “presence or absence of atheroma plaque” and the quantitative variable “number of teeth.”

**Results:**

A 6.2% prevalence of atheroma plaques was obtained from 1,079 selected X-rays. The risk in patients with periodontal disease increased as periodontal disease worsened. The risk in patients with periodontal disease increased as periodontal disease worsened as follows: healthy patients vs. periodontal patients with less than 30% bone loss in radiography: RR 0.434, 95% CI 0.181–1.041, *p* = 0.053 healthy patients vs. patients with between 30%–60% bone loss: RR 0.177, 95% CI 0.075–0.418, *p* < 0.05 healthy patients vs. patients with more than 60% bone loss: RR 0.121, 95% CI 0.041–0.355, *p* < 0.05. Patients with calcifications on their orthopantomograms had a lower mean teeth number (20.9 teeth) compared to patients without calcifications (24 teeth), which was statistically significant, *t* (1077) = −3.125, *p* < 0.05.

**Conclusions:**

Orthopantomography can be considered a screening method to detect patients at increased cardiovascular risk who are referred for individualized study. It is important to continue research to know the real significance of these findings. Dentists should be aware of the importance of our work in our patients' systemic health.

## 1. Introduction

According to the National Health System (NHS), cardiovascular diseases are the leading cause of death in the population in Spain. In men, ischemic heart disease is the leading cause of death, but it is stroke in women [1].

Moreover, they are a problem not only because of their mortality but also because they are diseases with high morbidity, causing lifelong neurological sequelae representing an enormous expense for the public health system and their families as well. Stroke in Spain, according to the SEN (Spanish Society of Neurology), represents an average cost per patient per year of approximately 27,711 euros. This cost includes direct healthcare costs, direct non-health-care costs (informal and formal care and other costs), and indirect costs (work productivity loss) [2].

The cerebral vascular disease (CVD) incidence in our country is 187.4/100,000 inhabitants, with a prevalence of 1.7%. This prevalence increases to 6.4% in patients over 70 years of age. Furthermore, it is estimated that CVD-related deaths will increase by 39% in the next 20 years [2].

On the other hand, one of the most important handicaps regarding stroke is the detection of patients at higher risk. Among the risk factors are hypertension, hypercholesterolemia, overweight, advanced age, or habits such as smoking and a sedentary lifestyle [3].

Since atheroma plaque detection is carried out with very specific tests for patients with a known potential risk, finding a noninvasive diagnostic method that has the capacity to screen very large populations would mean an important advance in early detection and preventive measures' implementation [4, 5].

Around 20–30% of cerebral infarctions are considered to be related to carotid arteriosclerosis [4]. Arteriosclerosis is a chronic pathology characterized by lipids and fibrosis' accumulation in arterial walls. It is a focal phenomenon that mainly affects arteries such as the aorta, coronary, carotid, iliac, and femoral arteries [6].

As the pathogenic potential of the atheroma plaque depends very much on its composition, plaques with a large lipid core and a thin fibrotic layer with many macrophages are vulnerable while those with a thicker fibrotic layer and fewer macrophages are more stable plaques and, subsequently, they can be classified into vulnerable and stable [4, 6, 7].

According to the White Book on Oral Health published by the Spanish Dental Council (2020) with data on the state of oral health in Spain, 51% of Spaniards visit the dentist at least once a year.

On the other hand, orthopantomography (OPG) is a radiological technique that shows an overview of the dent maxillomandibular region, allowing us to observe the maxillary structures and attached parts of the neck and skull. It is most commonly used in daily clinical practice [8].

It must be taken into account that both hard and soft tissues may have a certain distortion degree that we must be aware of, as well as unclear superimpositions in OPGs [8].

In 1995, Friedlander was the first to talk about the possibility of detecting atheroma plaques in the carotid region (C3-C4) in OPGs. These are almost always fortuitous findings, and a differential diagnosis must also be made with other structures (Table 1) which could be the object of confusion [9, 10] or other pathological entities [10–12]. Other authors have also studied the prevalence of atheroma plaques in OPG and obtained the results shown here (Table 2).

Other studies relate the increased prevalence of this finding to the presence of other cardiovascular risk factors such as hypertension, hypercholesterolemia, smoking, or previous cardiovascular events [17, 18].

There is evidence that periodontal disease increases the risk of suffering a cardiovascular event [19, 20]. Periodontal disease produces a gradual horizontal bone loss that can be radiographically evaluated.

Tooth loss has been an assessed risk factor for different diseases; for example, the WHO considers oral health to be one of the criteria included in the top ten of human health. Regarding the risk of suffering cardiovascular pathology or increasing mortality, the loss of two teeth is considered to increase the possibility of suffering cardiovascular disease by 3% [21].

The objectives of this study were to know the incidence of calcified atheroma plaques' appearance in orthopantomography and to analyze the relationship of calcified atheroma plaques with periodontal disease and missing teeth.

## 2. Material and Methods

The study analyzed the orthopantomographs of 1,254 patients over 18 years of age and performed at Clínica Arlanza, Lerma, Burgos, Spain, from April 2017 to June 2021. All X-rays were performed with the same orthopantomograph ProOne® (Planmeca, Helsinki Finland) with the parameters of 68 kV, 7 mA, and 10 seconds exposure and a single operator. Radiographic images were processed and visualized with Romexis® software (Planmeca, Helsinki Finland). This program allows modifying parameters such as brightness, contrast, and sizes to visualize areas in detail. All images were analyzed by a single observer trained in the detection and discrimination of images in the carotid region.

**Figure 1:**
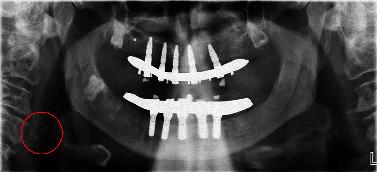
Typical location of atheroma plaque in orthopantomography. (Own source). In the image, we can observe a calcified lesion in the carotid region in front of C3-C4 following the anatomy of the artery.

Criteria related to image quality were considered during the evaluations. The reliability of the assessments was verified through a systematic process, which may have involved inter-rater reliability checks, calibration sessions, or consensus meetings among observers. Additionally, the observer had the capability to utilize image enhancement tools such as adjustments to brightness and contrast to optimize the visual clarity of the images during the evaluation process (Figure 1)

The inclusion and exclusion criteria were as follows:

- Inclusion criteria: patients, who attended Clínica Arlanza between 2017 and 2021, aged 18 or older, possessing an accessible primary care medical report, and having an orthopantomogram (OPG) with the C3-C4 zone visible and without artifacts. The image must be clearly visible.
- Exclusion criteria: individuals under 18 years of age, those with an unavailable OPG or medical report, or cases where the C3-C4 zone is not visible in the OPG, with the presence of artifacts or other circumstances preventing clear observation of the area.

As for the variables, they were selected based on the evidence relating them to CVD:

(i) Periodontal Disease: Patients were categorized into four groups based on their degree of bone loss:
  - Group I: no bone loss
  - Group II: <30% loss
  - Group III: 30–60% loss
  - Group IV: >60% loss
(ii) Remaining Teeth: Only erupted permanent or deciduous teeth were considered, regardless of their restorative status or whether they supported fixed prostheses. Included in the count were teeth without concern for their situation, excluding impacted teeth, implant-supported prostheses, or pontics of fixed units.

The radiographic appearance of a calcified atheroma, often observed in imaging studies like orthopantomograms (OPG) or dental X-rays, typically presents as a dense, well-defined, and opaque area within the vascular structure. Atheroma is the deposit of calcium, cholesterol, and other substances that accumulate on the inner lining of arteries over time.

This study complies with the Declaration of Helsinki (2013) and the provisions of the General Health Law on research. It has been approved by the Research Ethics Committee at European University Miguel de Cervantes. In addition, it protects confidentiality, and informed consent is collected from each participant.

The statistical analysis was conducted using SPSS Statistics® version 29. Results for categorical variables were presented as frequencies (%), and quantitative variables were described using the mean and standard deviation. Contingency tables, the chi-square test, or Fisher's exact test were employed to compare two categorical variables. The relative risk (RR) with its 95% confidence interval was used as a measure of statistical association.

## 3. Results

1,254 X-rays were analyzed and 175 were discarded because they did not meet inclusion criteria. 1,079 OPGs were selected in total. The distribution by sex was 581 women (53.8%) and 498 men (46.20%), with a mean age of 52.58 years.

Findings compatible with carotid atheromatous plaques were found in 67, which is 6.2% of the total. The distribution by gender was 6.2% for both genders. The mean age in the group with calcified lesions was 60.9 years and 52 years without calcified lesions.

The distribution of periodontal disease and remaining teeth in the sample are shown in Tables 3 and 4.

**Figure 2:**
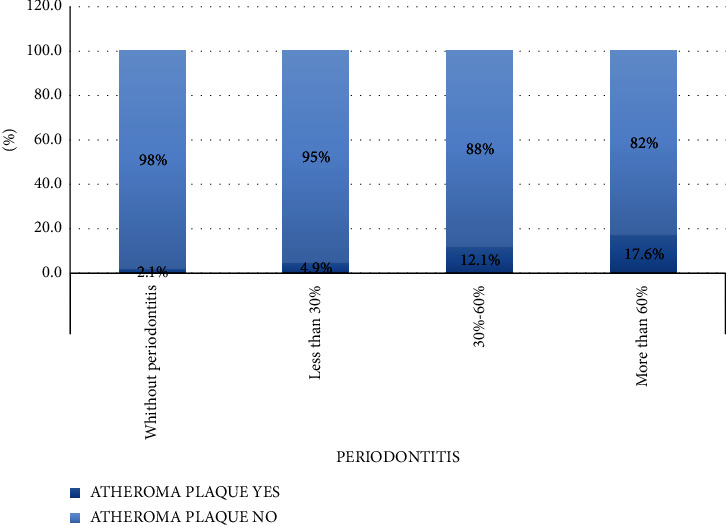
Atheroma plaques' prevalence in OPG according to periodontal disease severity.

The risk of finding calcified lesions in patients with periodontitis increases as periodontal disease worsens (Figure 2)

Healthy patients vs. patients with less than 30% bone loss: RR 0.434; 95% CI 0.181–1.041; *p*=0.053 although the result is not statistically significant. Healthy patients vs. patients with between 30%–60% bone loss: RR 0.177; 95% CI 0.075–0.418; *p* < 0.05 with a statistically significant result. Healthy patients vs. patients with more than 60% bone loss: RR 0.121; 95% CI 0.041–0.355; *p* < 0.05 with a statistically significant result.

Regarding the number of teeth, in the case of patients with calcifications in OPG, we found a lower mean number of teeth (20.9 teeth) than in patients without calcifications (24 pieces) being these data statistically significant, *t *(1077) = −3.125, *p* < 0.05.

## 4. Discussion

The prevalence of atheromatous plaques in OPG was 6.2%, which is in line with that published in scientific literature in which the results for prevalence in the general population are between 3% and 6.9% [11, 13, 15, 16, 22], and only Barona [17] has a much higher prevalence, 15.4%, probably due to the fact that his sample was collected in a hospital environment where other pathologies that are risk factors in the formation of atheroma plaques are more frequent.

However, our sample ranged from 18 to 98 years of age, in contrast to other studies where analysis begins above the age of 40 years [13–15]. This decision corresponds to the fact that the process of carotid plaque formation begins in postadolescence, so that focusing on more mature age groups may incur a bias [6].

These lesions' detection is certainly complex. The operator must be trained. In fact, there are authors who have implemented general dentists' capabilities with a two-week training when discerning these pathologies from others [23].

On the other hand, other complementary tests such as CBCT, where calcified atheroma plaques are also detected, are becoming more and more frequent [24, 25] being this an interesting line of research.

Naturally, the presence of calcified atheroma plaques in an orthopantomogram (OPG) does not necessarily indicate a direct correlation with an elevated stroke risk. Stroke is a multifactorial disease, not solely dependent on vessel lumen stenosis. Establishing a causal relationship is complex. However, it is essential to note that plaque rupture is a significant contributor to strokes, accounting for 20–30% of cases [7].

It is noteworthy that most studies [9, 11, 13–15] do not assess the correlation of plaque presence in orthopantomograms (OPG) with more specific diagnostic methods. Atalay [16] stands out as the exception, as they investigated patients with previously detected possible plaques in OPG (94 out of 1,650 patients) using Doppler methods. They confirmed the presence of plaques in 59 cases, while 34 cases showed no presence. Despite this, the author views it as a screening method worth considering, emphasizing the importance of considering other variables such as age, hypertension, and diabetes.

To truly establish the real risk, it would be interesting to analyze patients with plaques in OPG using appropriate methods such as PET, MRI, Doppler, or computed tomography angiography. There is literature evidence indicating a correspondence between findings in OPG and those observed in Doppler or angiography [26].

In another sense, other authors selected samples with previous pathologies that are known risk factors for stroke such as diabetes, hyperparathyroidism, previous stroke, or menopause, obtaining much higher results, between 20–40%. Therefore, analyzing these patients in greater detail is undoubtedly interesting considering these results [18, 26].

There is increasing evidence of periodontal disease's influence on systemic health, relating it to diabetes mellitus, preterm birth, Alzheimer's disease, and cardiovascular disease [27]. As for cardiovascular diseases, it is related in two ways [19, 20, 28].

- Direct: periodontal pathogens reach atheroma plaques by transient bacteremia destabilizing them and causing a cardiovascular event
- Indirect: periodontal disease generates chemical mediators of inflammation, such as IL-1, IL-6, TNG-a, or C-reactive protein, that reach the systemic circulation and, in sufficient levels and with other circumstances, could be capable of destabilizing atheroma plaques and producing a cardiovascular event

There are authors who even relate periodontal disease with atheroma plaques' detection in OPG as a risk factor for suffering a cardiovascular event, in this case, myocardial infarction [29]. Of course, teeth absence is difficult to dissociate from periodontal disease itself, although, as mentioned above, it is an independently related factor to cardiovascular disease [21].

Results show that atheromatous plaques' presence increases significantly with periodontal disease and with its severity, from 2.1% prevalence in healthy patients to 17.6% in patients with more than 60% bone loss. When periodontal health is compared with periodontal disease in all cases, we find that being periodontally healthy is a protective factor for atheromatous plaques' appearance in OPG.

Despite the results of this study, the diagnosis of the presence of atheromatous plaque should not rely solely and exclusively on orthopantomography. This test should be considered a red flag leading to other investigations, including blood tests with cholesterol levels, and examinations of other imaging modalities.

Likewise, the data from the sample regarding teeth loss also show that patients with atheroma plaques have on average fewer teeth (20.9 teeth) compared to those without plaques (24 teeth).

Further research should be carried out in this line to establish a greater correlation between image and real risk, in addition to analyzing various variables of the patient's general or oral health. Dentists should have a more global vision of their patients to be able to see beyond the mouth. They should know that there are oral pathologies that have systemic implications, and they should be trained in these lesions' detection with a clear referral protocol to reference centers.

## 5. Conclusions

In conclusion, discovering calcifications in an orthopantomogram (OPG) that may correspond to carotid atheroma plaques does not guarantee that a patient will experience a stroke. However, it is crucial not to disregard such findings and focus solely on dental issues.

This situation merits careful analysis, as, when combined with a thorough clinical history and proper anamnesis, it can lead to specialized care that unveils a silent cardiovascular problem. It is essential to emphasize that early diagnosis of cardiovascular diseases is critical.

Therefore, if a minimally invasive test like OPGs can assist in identifying patients unaware of their cardiovascular risk, dentists could play an active role in promoting cardiovascular health. This complements the evolving role of dentists in periodontal medicine, contributing to the improvement of the patient's systemic condition through the control of periodontal disease.

## Figures and Tables

**Table 1:** Anatomical and pathological structures that can be confused with atheroma plaques in orthopantomography.

Anatomical structure	Pathological entity
Hyoid bone	Calcified lymph nodules
Stylohyoid process	Phlebitis
Stylohyohyoid ligament calcification	Sialoliths of the submandibular gland
Stylomandibular ligament calcification	Foreign bodies
Thyroid cartilage	Thyroid gland calcifications
Triquetrum cartilage	
Epiglottis	

**Table 2:** Authors studying atheromatous plaques' prevalence in the general population.

Author	Year	Sample	Age	Prevalence (%)
Friedlander and De Bake [9]	1994	295 patients	55–84 years old	3
Carter [13]	1997	1,175 patients	>40 years old	3.6
Cohen et al. [14]	2002	71 patients	>55 years old	3.8
Bayer et al. [11]	2011	2,557 patients	>30 years old	5
Atalay et al. [15]	2015	1,650 patients	>45 years old	5.6
Gonçalves et al. [16]	2016	8,338 patients	4–94 years old	6.9
Barona-Dorado et al. [17]	2016	1,130 patients	>18 years old	15.4

**Table 3:** Periodontal disease distribution in the sample.

	No BL	<30% BL	30–60% BL	>60% BL
Patients (*n*)	280	530	240	34
Sample (%)	25.8	48.9	22.1	3.1

BL: bone loss.

**Table 4:** Remaining teeth distribution in the sample.

	Patients (*n*)	Sample (%)
0 teeth	24	2.2
1 tooth	5	0.5
2 teeth	6	0.6
3 teeth	3	0.3
4 teeth	7	0.6
5 teeth	7	0.6
6 teeth	12	1.1
7 teeth	8	0.7
8 teeth	10	0.9
9 teeth	9	0.8
10 teeth	10	0.9
11 teeth	11	1
12 teeth	14	1.3
13 teeth	6	0.6
14 teeth	13	1.2
15 teeth	15	1.4
16 teeth	13	1.2
17 teeth	19	1.8
18 teeth	18	1.7
19 teeth	24	2.2
20 teeth	38	3.5
21 teeth	26	2.4
22 teeth	26	2.4
23 teeth	27	2.5
24 teeth	50	4.6
25 teeth	62	5.7
26 teeth	61	5.6
27 teeth	66	6.1
28 teeth	168	15.5
29 teeth	81	7.5
30 teeth	92	8.5
31 teeth	62	5.7
32 teeth	90	8.3
33 teeth	1	0.1
Total	1,079	100%

## Data Availability

The datasets generated during and/or analyzed during the current study are available from the corresponding author on reasonable request.

## References

[1] Bueno H., Pérez-Gómez B. (2019). Global rounds: cardiovascular health, disease, and care in Spain. *Circulation*.

[2] Sen (2019). *GdEdECdlSEdN. Atlas del Ictus España 2019*.

[3] Portegies M. L., Koudstaal P. J., Ikram M. A. (2016). Cerebrovascular disease. *Handbook of Clinical Neurology*.

[4] Fuster V. (2001). Avances en el diagnóstico por resonancia magnética de la enfermedad arterial. *Revista Española de Cardiología*.

[5] Gokaldas R., Singh M., Lal S., Benenstein R. J., Sahni R. (2015). Carotid stenosis: from diagnosis to management, where do we stand?. *Current Atherosclerosis Reports*.

[6] Bartomeu-Ruiz A., Zambón-Rados D. (2002). La placa aterogénica: fisiopatología y consecuencias clínicas. *Medicina Integral*.

[7] Rognoni A., Cavallino C., Veia A. (2015). Pathophysiology of atherosclerotic plaque development. *Cardiovascular and Hematological Agents in Medicinal Chemistry*.

[8] Cosson J. (2020). Interpreting an orthopantomogram. *Australian Journal of General Practice*.

[9] Friedlander A. H., De Baker J. N (1994). Panoramic radiography: an aid in detecting patients at risk of cearebrovascular accident. *The Journal of the American Dental Association*.

[10] Friedlander A. H. (1995). Panoramic radiography: the differential diagnosis of carotid artery atheromas. *Special Care in Dentistry*.

[11] Bayer S., Helfgen E. H., Bös C., Kraus D., Enkling N., Mues S. (2011). Prevalence of findings compatible with carotid artery calcifications on dental panoramic radiographs. *Clinical Oral Investigations*.

[12] Carter L. C. (2000). Discrimination between calcified triticeous cartilage and calcified carotid atheroma on panoramic radiography. *Oral Surgery, Oral Medicine, Oral Pathology, Oral Radiology & Endodontics*.

[13] Carter L. C., Haller A. D., Nadarajah V., Calamel A. D., Aguirre A. (1997). Use of panoramic radiography among an ambulatory dental population to detect patients at risk of stroke. *The Journal of the American Dental Association*.

[14] Cohen S. N., Friedlander A. H., Jolly D. A., Date L. (2002). Carotid calcification on panoramic radiographs: an important marker for vascular risk. *Oral Surgery, Oral Medicine, Oral Pathology, Oral Radiology & Endodontics*.

[15] Atalay Y., Asutay F., Agacayak K. S., Koparal M., Adali F., Gulsun B. (2015). Evaluation of calcified carotid atheroma on panoramic radiographs and Doppler ultrasonography in an older population. *Clinical Interventions in Aging*.

[16] Gonçalves J. R., Yamada J. L., Berrocal C., Westphalen F. H., Franco A., Fernandes Â (2016). Prevalence of pathologic findings in panoramic radiographs: calcified carotid artery atheroma. *Acta Stomatologica Croatica*.

[17] Barona-Dorado C., Gutierrez-Bonet C., Leco-Berrocal I., Fernández-Cáliz F., Martínez-González J. M. (2016). Relation between diagnosis of atheromatous plaque from orthopantomographs and cardiovascular risk factors. A study of cases and control subjects. *Med Oral Patol Oral Cir Bucal*.

[18] Gustafsson N., Ahlqvist J. B., Näslund U., Wester P., Buhlin K., Gustafsson A. (2018). Calcified carotid artery atheromas in panoramic radiographs are associated with a first myocardial infarction: a case-control study. *Oral Surgery, Oral Medicine, Oral Pathology, and Oral Radiology*.

[19] Liccardo D., Cannavo A., Spagnuolo G., Ferrara N., Cittadini A., Rengo C. (2019). Periodontal disease: a risk factor for diabetes and cardiovascular disease. *International Journal of Molecular Sciences*.

[20] Sanz M., Del Castillo A. M., Jepsen S., Gonzalez-Juanatey J. R., D’Aiuto F., Bouchard P. (2020). Periodontitis and cardiovascular diseases. Consensus report. *Glob Heart*.

[21] Cheng F., Zhang M., Wang Q., Xu H., Dong X., Gao Z. (2018). Tooth loss and risk of cardiovascular disease and stroke: a dose-response meta analysis of prospective cohort studies. *PLoS One*.

[22] Ohba T., Takata Y., Ansai T., Morimoto Y., Tanaka T., Kito S. (2003). Evaluation of calcified carotid artery atheromas detected by panoramic radiograph among 80-year-olds. *Oral Surgery, Oral Medicine, Oral Pathology, Oral Radiology & Endodontics*.

[23] Gustafsson N., Ahlqvist J., Levring Jäghagen E. (2019). Long-term skill improvement among general dental practitioners after a short training programme in diagnosing calcified carotid artery atheromas on panoramic radiographs. *European Journal of Dental Education*.

[24] Soares A. D., Wanzeler A. M., Oliveria Renda M. D., Marinho C. G., Tuji F. M. (2017). Cone-Beam computed Tomography findings in the early diagnosis of calcified atheromas. *Journal of Oral and Maxillofacial Surgery*.

[25] Tanabe J., Tanaka M., Kadooka K., Hadeishi H. (2016). Efficacy of high-resolution cone-beam CT in the evaluation of carotid atheromatous plaque. *Journal of Neurointerventional Surgery*.

[26] Friedlander A. H., Aghazadehsanai N., Chang T. I., Harada N., Garrett N. R. (2013). Prevalence of calcified carotid artery atheromas on panoramic images of individuals with primary hyperparathyroidism. *Dentomaxillofacial Radiology*.

[27] Beck J. D., Papapanou P. N., Philips K. H., Offenbacher S. (2019). Periodontal medicine: 100 Years of progress. *Journal of Dental Research*.

[28] Joshi C., Bapat R., Anderson W., Dawson D., Hijazi K., Cherukara G. (2021). Detection of periodontal microorganisms in coronary atheromatous plaque specimens of myocardial infarction patients: a systematic review and meta-analysis. *Trends in Cardiovascular Medicine*.

[29] Gustafsson N., Ahlqvist J., Näslund U. (2020). Associations among periodontitis, calcified carotid artery atheromas, and risk of myocardial infarction. *Journal of Dental Research*.

